# Modeling the blood–brain barrier using stem cell sources

**DOI:** 10.1186/2045-8118-10-2

**Published:** 2013-01-10

**Authors:** Ethan S Lippmann, Abraham Al-Ahmad, Sean P Palecek, Eric V Shusta

**Affiliations:** 1 Dept. of Chemical and Biological Engineering, University of Wisconsin-Madison, 1415 Engineering Dr., Madison, WI, 53706, USA

**Keywords:** Blood–brain barrier, Pluripotent stem cell, Neural progenitor cell

## Abstract

The blood–brain barrier (BBB) is a selective endothelial interface that controls trafficking between the bloodstream and brain interstitial space. During development, the BBB arises as a result of complex multicellular interactions between immature endothelial cells and neural progenitors, neurons, radial glia, and pericytes. As the brain develops, astrocytes and pericytes further contribute to BBB induction and maintenance of the BBB phenotype. Because BBB development, maintenance, and disease states are difficult and time-consuming to study *in vivo*, researchers often utilize *in vitro* models for simplified analyses and higher throughput. The *in vitro* format also provides a platform for screening brain-penetrating therapeutics. However, BBB models derived from adult tissue, especially human sources, have been hampered by limited cell availability and model fidelity. Furthermore, BBB endothelium is very difficult if not impossible to isolate from embryonic animal or human brain, restricting capabilities to model BBB development *in vitro*. In an effort to address some of these shortcomings, advances in stem cell research have recently been leveraged for improving our understanding of BBB development and function. Stem cells, which are defined by their capacity to expand by self-renewal, can be coaxed to form various somatic cell types and could in principle be very attractive for BBB modeling applications. In this review, we will describe how neural progenitor cells (NPCs), the *in vitro* precursors to neurons, astrocytes, and oligodendrocytes, can be used to study BBB induction. Next, we will detail how these same NPCs can be differentiated to more mature populations of neurons and astrocytes and profile their use in co-culture modeling of the adult BBB. Finally, we will describe our recent efforts in differentiating human pluripotent stem cells (hPSCs) to endothelial cells with robust BBB characteristics and detail how these cells could ultimately be used to study BBB development and maintenance, to model neurological disease, and to screen neuropharmaceuticals.

## Review

### Blood–brain barrier development and maintenance

**Figure 1:**
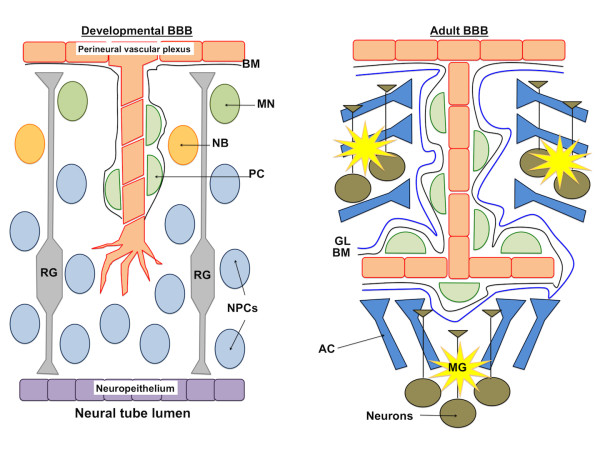
**Schematic representation of the developmental and adult BBB.** Embryonic blood vessels invade the neural tube by the migration of the tip cell towards the neuroepithelium. Newly forming blood vessels actively recruit pericytes (PC) that ensure the stabilization of the new structure and synthesize an embryonic basement membrane (BM). In parallel to cerebral angiogenesis, neural progenitor cells (NPCs) originating from the neuroepithelium start to migrate towards the upper layers of the cerebral cortex using radial glia (RG) as a guidance structure. During their migration, these NPCs begin differentiation into neuroblasts (NB) and maturing neurons (MN). In contrast to the developmental BBB, the adult BBB constitutes a more elaborate structure. The cerebral vasculature shares a BM with PCs. The BM is more complex and is surrounded by an external tunica, the glia limitans (GL). The BM and the GL are separated by a perivascular space. On the outer side of the GL, blood vessels are highly invested by astrocyte end-feet processes (AC) and surrounded by neurons and microglial cells (MG). Neurons may directly and indirectly interact with the cerebral vasculature.

In order to appreciate the potential impact for stem cell modeling of the BBB, it is useful to briefly review the processes of BBB formation and maintenance. Unlike other tissues, central nervous system (CNS) vascularization is exclusively driven by angiogenesis. In rodents, cerebral blood vessels are formed around embryonic day 9 (E9) by sprouting from the perineural vascular plexus (PNVP) [1], a primitive vascular network surrounding the neural tube (Figure 1). Under the influence of vascular endothelial derived growth factor (VEGF), Angiopoietin-1, and sonic hedgehog (Shh) secreted by the neuroepithelium lining the subventricular zone [2], certain endothelial cells (ECs) of the PNVP switch their phenotype to tip cells, a highly invasive and migratory EC type that initiates blood vessel sprouting into the neural tube. Differentiating brain endothelial cells are anchored on a primitive basement membrane (BM) formed by various extracellular matrix (ECM) proteins including collagen IV, fibronectin, laminin-1 and entactin/nidogen-1 [3-5]. Also, the rapid coverage of such newly formed microvasculature by pericytes suggests that they may be the first cell type of the neurovascular unit to physically interact with endothelial cells [5]. In addition to pericytes, neighboring undifferentiated neural progenitor cells (NPCs), differentiating NPCs, and radial glia also appear to exercise an influence on the developmental BBB as studies have suggested their ability to induce barrier properties in brain endothelial cells *in vitro* and *in vivo*[6-9]. On the other hand, the early stage developing brain vasculature remains devoid of astrocytes as such cells only appear at the end of gestation and early postnatal stages [10,11]. While the nature of the molecular signals imparted on the brain endothelial cells by the neighboring cells of the developing neurovascular unit remains unclear, recent studies have highlighted the importance of Wnt signaling (through the secretion of Wnt7a/Wnt7b, likely by NPCs), GPR124 and Shh [6,12-18]. During embryonic development, functional barrier properties are acquired as demonstrated by a continuous increase in tight junction (TJ) organization [19,20]. This process results in barrier maturation, marked by an increase in transendothelial electrical resistance (TEER) from < 500 Ωxcm^2^ to ~1500 Ωxcm^2^[21] with a concomitant decrease in permeability to water-soluble compounds such as mannitol, potassium or urea [22,23].

Although barrier properties are certainly induced during embryonic development, they remain attenuated when compared to the adult BBB. An examination of the multicellular composite that helps maintain the adult BBB reveals that pericytes remain in contact with ECs, sharing a more elaborate BM formed by different ECM components including agrin, laminin, perlecan and SPARC/osteonectin (Figure 1). The developmental brain parenchyma is replaced by a densely populated neuropil formed by neurons and glial cells supported by a chondroitin-sulfate proteoglycan-rich matrix [24]. Unlike the early stages of embryonic BBB development when astrocytes are absent, astrocytes play important roles in BBB maturation and maintenance. As a result of this adult brain microenvironment and in contrast to the developmental BBB, the adult BBB boasts an elevated TEER, measured at average values between 1000–2000 Ωxcm^2^ (and maximum values up to 6000 Ωxcm^2^) and a correspondingly lower passive permeability to molecular tracers [21,25,26]. These mature brain endothelial cells also express a broad array of large and small molecule transport systems including nutrient influx transporters and efflux transporters such as p-glycoprotein (p-gp), multi-drug resistance-associated proteins (MRP), and breast cancer resistance protein (BCRP) (for a review, see [27]). While the mechanisms driving the further induction and maintenance of the adult BBB are unresolved, several growth factors and signaling molecules such as angiopoietin-1 [28], cyclic adenosine monophosphate [29], basic fibroblast growth factor [30], glial-derived neurotrophic factor [31], glucocorticoids [32,33], retinoic acid [30], src-suppressed C kinase substrate [34], Shh [14], transforming growth factor β [35] and Wnt3a [13] have been shown to have effects on the BBB phenotype *in vitro*. Importantly, the BBB phenotype is dictated by the local microenvironment and is not intrinsic to brain endothelial cells themselves [36]; and thus, primary brain microvascular endothelial cells (BMECs) rapidly lose their barrier features *in vitro*. When modeling the BBB, as discussed in the upcoming section, it is important to take into account the microenvironment that needs to be recreated with the embryonic and adult neurovascular units comprising very different cellular and molecular architectures.

### In vitro modeling of the BBB

Modeling the BBB *in vitro* can facilitate a variety of studies that are not amenable to *in vivo* investigation. For example, *in vivo* experiments, such as those performed with knockout animals, are largely restricted to evaluating basic phenotype alterations, resulting in a limited understanding of underlying molecular and cellular mechanisms that may govern a physiological process or BBB dysfunction in a disease state. Also, while detailed drug delivery evaluation can only be performed *in vivo*, mining through large combinatorial libraries of small molecule or protein libraries is not compatible with *in vivo* approaches. Finally, *in vivo* investigation of the BBB is mostly performed in animals, with investigation of the human BBB being limited to non-invasive methods such as magnetic resonance imaging techniques.

**Figure 2:**
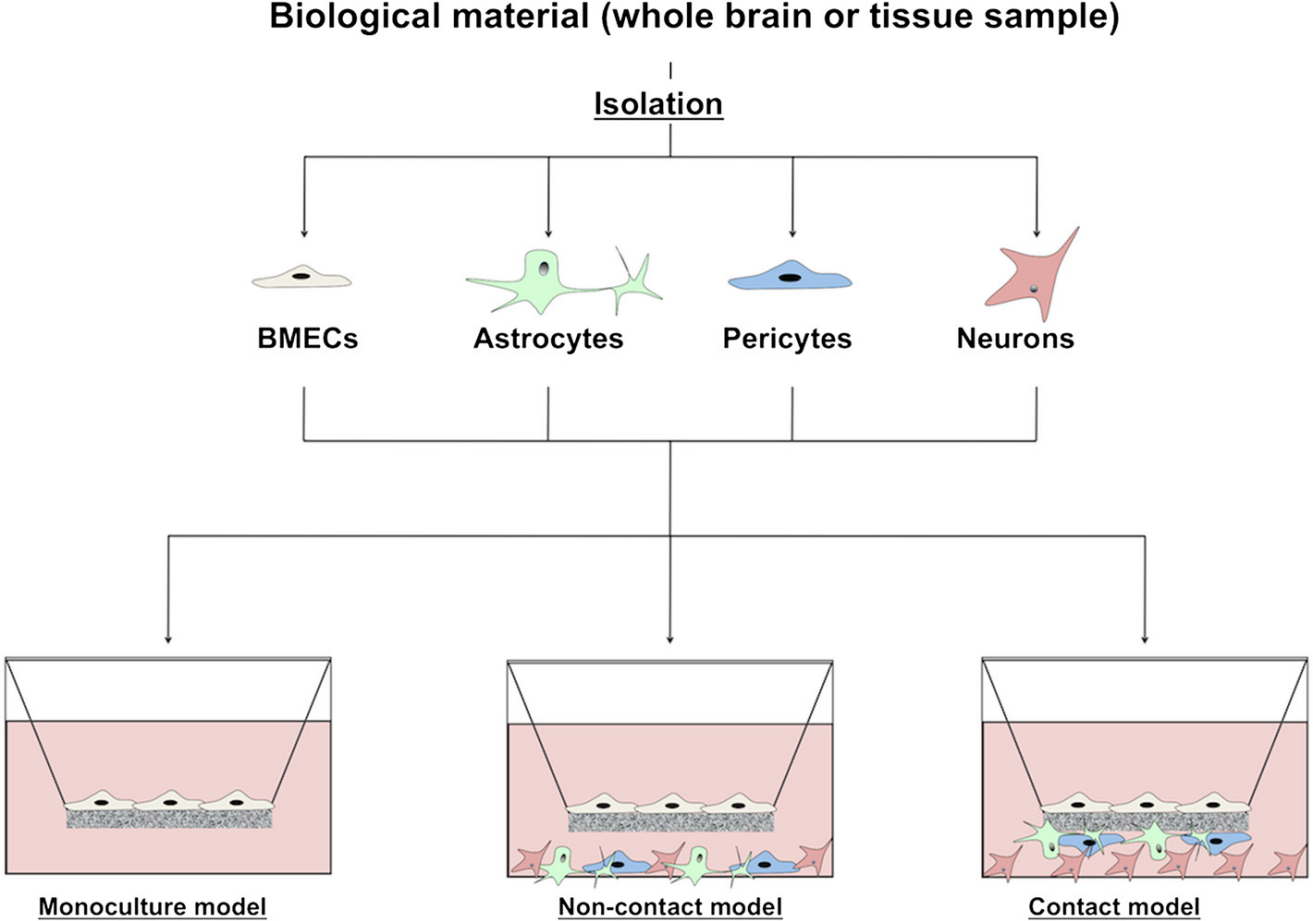
**Schematic representation of the various BBB *in vitro *models.** Cells are isolated from whole brain tissue (non-human origin) or from biopsied tissue samples (human origin). From these sources, primary cultures of BMECs, astrocytes, pericytes and neurons can be achieved. In the case of BMECs, immortalized cells lines have been established from both rodent (bEnd.3, RBE4) and human (hCMEC/D3) cells. Cells can be cultivated in either a BMEC monoculture or in a co-culture model including any combination of astrocytes, pericytes and neurons. Co-cultures can be established in a non-contact manner or in a contact manner by seeding a co-cultured cell on the other side of the filter.

Because of the significant challenges presented by *in vivo* studies, *in vitro* models have been under development and utilized in countless scientific studies (Figure 2). One longstanding approach consists of isolating and culturing primary BMECs. Given the aforementioned complex intercellular interplay that defines the embryonic and adult neurovascular unit, one can imagine that removal of BMECs from their brain microenvironment and growth in culture can lead to loss of BBB phenotype. To date, there has been very limited success in coaxing embryonic BMECs to grow *ex vivo*[37]. On the other hand, adult BMECs have been cultured successfully by many laboratories, but they rapidly lose their *in vivo* phenotype resulting in comparatively poor TEER (100–200 Ωxcm^2^), high paracellular permeability (~100x higher than the *in vivo* situation) and decreased transporter expression compared to the same cells *in vivo*[38-40]. In addition, given that brain vasculature comprises only 0.1% of the brain by volume, such techniques require a significant amount of brain material to achieve a reasonable yield of BMECs, limiting high throughput applications. A seemingly inviting, scalable alternative is the use of immortalized brain endothelial cell lines. Examples of widely used brain endothelial cell lines described in the literature include the immortalized hCMEC/D3 human cell line [41], the rat RBE4 cell line [42] and the mouse bEnd.3 cell line [43]. The main advantage of such cell lines is the expansion capacity derived from their immortalized status. However, while these cell lines maintain many aspects of their primary BMEC counterparts and represent very useful tools for certain applications, they lack significant barrier function [44,45].

In order to improve primary BMEC properties, various approaches to re-introduce aspects of the *in vivo* microenvironment have been reported. Astrocyte co-culture systems are the most widely used [46,47]. In this model, BMECs are cultivated, usually in a non-contact format, with primary astrocytes isolated from newborn rodents (Figure 2). Addition of astrocytes can improve barrier function as measured by increases in TEER and decreases in passive permeability [47-50]. Following the isolation and characterization of adult brain pericytes by Dore-Duffy and colleagues [51], several studies highlighted the ability of primary pericyte co-cultures to improve barrier function. Finally, by comparison, the impact of neurons on barrier function *in vitro* appears lessened compared with astrocytes and pericytes [52-55]. Co-culture with each of these cell types alone has been reported to increase TEER [47,56] and decrease paracellular permeability [47,52,56]. Such improved barrier properties involved enhancement of TJ complexes as observed by increased protein levels as well as an enhanced localization [46,49,53,55,57,58]. In addition to improved barrier phenotype, several studies also reported an enhanced efflux transporter activity, in particular that mediated by p-gp [56,59]. Comparatively, astrocytes co-cultures appear to have better induction on barrier properties and TJ complexes formation than pericytes as noted by different studies [58,60,61]. However such studies also noted a partial additive effect *in vitro* when BMECs were co-cultured simultaneously with astrocytes and pericytes [60,61] (Figure 2), suggesting that these cell types may use common signaling pathways or act synergistically to induce barrier properties in BMECs, while also inducing some cell-specific signaling pathways. In addition to conventional 2-dimensional co-cultures models, different *in vitro* BBB models have been developed in the last decade using natural (collagen, hydrogel) or synthetic materials (polypropylene) to obtain a 3-dimensional scaffold structure [62-65]. These models demonstrate the effects of two-dimensional co-culture, three-dimensional co-culture, or continuous laminar shear stress on BMEC morphogenesis and barrier-genesis.

Although the BBB properties of such multicellular co-culture models have improved as a result of the synergistic combination of the various cell types of the neurovascular unit, these models still fail to fully recreate the *in vivo* BBB phenotype. In addition, implementation of such models is limited by two factors: workflow and scalability. Neurons (embryonic), astrocytes (postnatal), pericytes (adult), and BMECs (adult) are isolated from animals of various ages, resulting in a laborious process of many singular primary cell isolations, and yields from several of these isolations, particularly of BMECs, are quite low. Finally, although cellular cross-talk can be observed between BBB cells from different species [47,66], mixed species co-cultures might remain suboptimal compared to syngeneic co-cultures. Because such syngeneic co-cultures remain limited to rodent BBB models, it would be useful to have a new approach to obtain an all-human *in vitro* BBB model.

### Stem cells sources for BBB modeling

**Figure 3:**
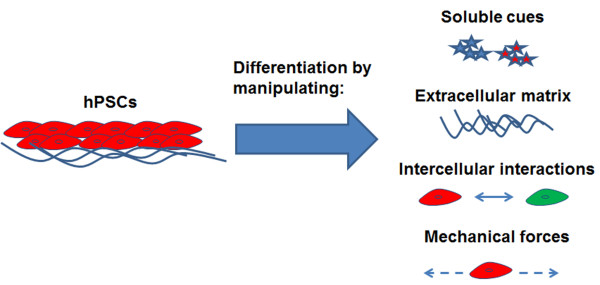
**Methods for differentiating hPSCs.** hPSCs can be differentiated to many different somatic cell types by manipulating a variety of conditions. Soluble cues, including growth factors and small molecules, can activate or inhibit signaling pathways to help direct cell fate. Extracellular matrix composition can also influence cell fate. Autocrine, paracrine, or juxtacrine signaling between neighboring cells can substantially affect differentiation outcomes. Mechanical forces can also be applied to guide hPSC differentiation.

A stem cell-based paradigm has the potential to offer substantial advantages for BBB modeling because of the current challenges with multicellular complexity, scalability, human sourcing, and the inability to culture primary BMECs at different developmental time points, particularly early in embryonic development. As a brief background, a stem cell is generally defined by its capacity for extensive self-renewal and ability to generate terminal progeny. In broad terms, stem cells give rise to all cells in the human body throughout various stages of development and then often reside in specific locations, or niches, during adulthood, such as in the subventricular zone and the hippocampal dentate gyrus of the brain [67-69] and the hematopoietic stem cells in the bone marrow [70]. Various populations of stem cells can be isolated during development and from adult tissues, and the properties they possess are dependent on the timing and location of the isolation. Embryonic stem cells (ESCs), which are derived from the inner mass of blastocyst-stage embryos, are termed pluripotent because they can form somatic cells from all three primitive germ layers (ectoderm, endoderm, and mesoderm) [71-73]. Stem cell populations with more restricted fate potential, including most adult stem cells, are termed multipotent. For instance, neural progenitor cells (NPCs) isolated from the embryonic CNS can differentiate into neurons, astrocytes, and oligodendrocytes [74,75]. Somatic cells can also be reprogrammed to a pluripotent state (induced pluripotent stem cells; iPSCs) or multipotent state (e.g. induced neural stem cells) via forced expression of various transcription factors regulating pluripotency [76-81]. These various types of stem cells, especially human ESCs (hESCs) and human iPSCs (hiPSCs), have enormous potential for the study of human development and disease. For instance, hPSCs have been differentiated into diverse cell types such as cardiomyocytes [82], beta-pancreatic cells [83], neurons and glia [84], retina [85], and even three-dimensional structures such as the optic cup [86], typically by directed manipulation of intracellular and extracellular signaling pathways via protein or small molecule treatments, intercellular interactions, mechanotransduction, or matrix-mediated cues [87] (Figure 3). These differentiation protocols allow access to cell populations, including transient developmental progenitors and terminally differentiated cells that would otherwise be unattainable from human tissue. hiPSCs can also be used to capture and study the phenotype of various genetic diseases [88] such as spinal muscular atrophy [89], Alzheimer’s disease [90], familial dysautonomia [91], and Rett syndrome [92] by isolating cells from a patient harboring the genetic disease, creating an iPSC line, and differentiating that line to the cell type(s) affected by the disease. hPSCs also offer significant utility for screening prospective therapeutics. Compounds screened in animals or against cell lines often fail in clinical trials due to toxicity or a lack of efficacy [93], which highlights the need for improved model systems for drug screening. Human PSCs have thus far gained traction for testing drugs for heart toxicity using hPSC-derived cardiomyocytes [94,95] and may be useful for other organs if the relevant hPSC-derived cell types adequately represent their *in vivo* counterparts.

The aforementioned properties of stem cells make them attractive candidates for modeling the BBB. Unlike primary cells, stem cells can be propagated extensively *in vitro* and because they can be derived from a clonal source, their progeny have a homogeneous genetic profile. Stem cells can also provide intermediate populations in development whereas mature cells isolated from adult tissue cannot. To apply stem cells to BBB modeling applications, the appropriate stem cell population must be selected. Namely, modeling BBB development requires cells with an embryonic phenotype, whereas modeling BBB maintenance and constructing a model for drug screening would require cells with a mature adult phenotype. To this end, we have utilized multiple stem cell sources in our laboratory for various BBB applications over the last several years. We first utilized NPCs to model aspects of BBB development and demonstrated that embryonic NPCs in the early stages of differentiation contribute to BBB properties *in vitro*[9]. We next utilized NPC-derived neurons and astrocytes having a more mature phenotype for modeling the adult BBB [66]. Finally, we have recently described a process to generate BMECs from hPSCs and monitor human BBB development in *vitro*[96]. Upon maturation, these BMECs may also be useful for drug screening applications. In this review, we will describe these efforts in detail, as well as outline the potential uses and concerns of each cell source to motivate future work.

## Stem cell modeling of the BBB

### Stem cell modeling of BBB development

**Figure 4:**
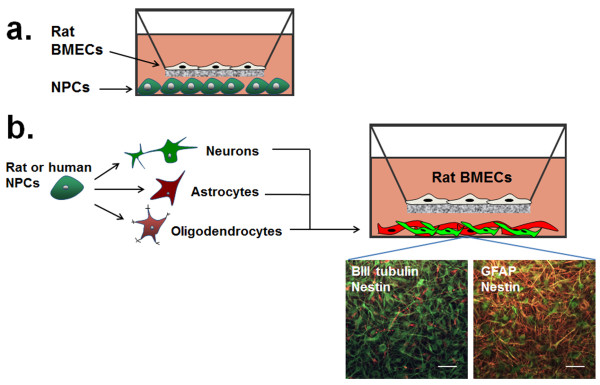
**Schematic representation of BMEC-NPC co-culture schemes. a)** NPCs were first utilized to examine non-contact interactions with rat BMECs. **b)** NPCs of rat and human origin were pre-differentiated to mixtures of neurons, astrocytes, and oligodendrocytes and co-cultured with rat BMECs. Human NPCs differentiated for 9 days yield progeny such as βIII tubulin^+^ neurons (left panel; red) and GFAP^+^ astrocytes (right panel; red) with extensive nestin expression (green). Scale bars indicate 50 μm.

As discussed, cell types other than astrocytes are likely responsible for the initial induction of BBB properties during embryonic development. To address this issue, our research group used embryonic NPCs along with primary BMECs as an *in vitro* model of the developmental BBB (Figure 4a) [9]. The purpose of this study was to isolate a population of rat cortical NPCs from embryonic day 14 (E14), corresponding to the timeframe when the BBB phenotype is induced *in vivo* but prior to astrocyte formation, and determine their capability for inducing BBB properties in cultured adult rat BMECs. The initial results from this study indicated that NPCs maintained in their undifferentiated state could not induce BBB properties in the cultured BMECs, but when NPCs in the early stages of differentiation were co-cultured with BMECs, the BMECs exhibit an increase in passive barrier properties as measured by elevated TEER and decreased permeability to the small molecule tracer sodium fluorescein. At an ultrastructural level, BMECs co-cultured with differentiating NPCs possessed a higher percentage of smooth and continuous tight junctions as determined by monitoring the localization of proteins such as claudin-5, occludin, and ZO-1. Analysis of the NPC-derived progeny revealed that differentiation in the presence of BMECs resulted in significantly more cells expressing nestin (a marker of immature neural progenitors) but fewer cells undergoing neuronal differentiation as measured by βIII tubulin expression, a similar finding to that shown previously using a mouse brain endothelial cell line in co-culture with NPC-derived cells [97]. Interestingly, if instead NPCs were differentiated for 24 hours in the absence of BMECs prior to co-culture, the mixture contained more βIII tubulin^+^ neurons and fewer nestin-expressing precursors, but the co-cultures were unable to substantially induce elevated BMEC TEER. Taken together, these results indicated that NPCs in their early stages of differentiation, likely in the nestin-expressing state, have the potential to induce BBB properties in BMECs, and do so in a manner distinct in timing and duration from postnatal astrocytes. Other researchers have confirmed the influence of NPCs on BBB character *in vitro*[98], and several studies have since linked BBB induction to Wnts supplied by the developing neural tube *in vivo*, identifying a potential link between the *in vitro* and *in vivo* effects of NPCs [6,8].

**Figure 5:**
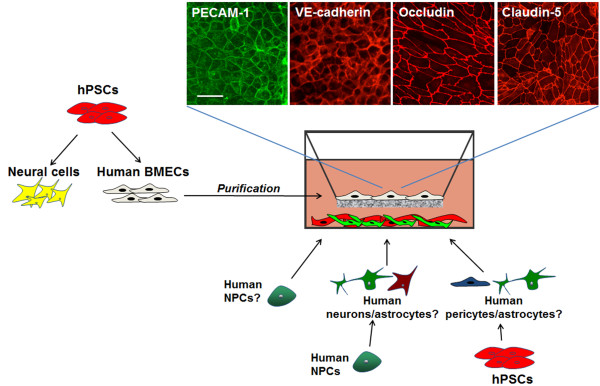
**Progress towards an all-human stem cell-derived *in vitro *BBB model.** hPSCs can be co-differentiated as a mixture of neural cells and BMECs, and the BMECs can be subcultured as a pure monolayer expressing typical endothelial and BBB markers such as PECAM-1, VE-cadherin, occludin, and claudin-5. Several options are theoretically possible for creating an all-human BBB model with these hPSC-derived BMECs. Human NPCs could potentially be used to create a BMEC/NPC co-culture model as a representative *in vitro* model of the developing human BBB. Alternatively, human NPCs could be pre-differentiated into mixed neuron/astrocyte cultures to model the adult BBB. Ideally, future applications will involve using hPSCs to obtain all the different cells forming the neurovascular unit. This approach could also facilitate the use of hiPSCs derived from both healthy and diseased patients to obtain a physiological or diseased model of the human BBB *in vitro*. Scale bar indicates 25 μm.

A limitation of the aforementioned developmental BBB model was the use of adult BMECs as opposed to embryonic BMECs. Thus, we next attempted to employ hPSCs to generate a more representative model of the developmental BBB in which brain endothelial inductive cues could be identified and systematically analyzed. While endothelial cells have previously been differentiated from hPSCs, they had not yet been shown to possess organ-specific phenotypes or gene expression signatures [99-101]. However, given the embryonic brain microenvironment comprising primitive endothelial cells and differentiating NPCs and our findings that differentiating NPCs could induce BBB properties, we hypothesized that co-differentiating neural cells could impart a BBB phenotype on hPSC-derived endothelium (Figure 5) [96]. To this end, we identified differentiation and culture conditions where hPSCs generate a co-differentiating mixture of primitive endothelium and NPCs. In this approach, a population of PECAM-1^+^ cells lacking tight junctions and mature endothelial cell markers such as von Willebrand Factor (vWF) and VE-cadherin was expanded within a mixed neural population predominantly comprised of nestin^+^/βIII tubulin^-^ progenitors and nestin^+^/βIII tubulin^+^ immature neurons. These neural populations expressed *WNT7A* and *WNT7B*, which are expressed by NPCs *in vivo*, and contribute to BBB development [6,8]. As the neural population matured into mainly nestin^+^/βIII tubulin^+^ and nestin^-^/βIII tubulin^+^ neurons, the endothelial cells began to express hallmark biomarkers of the BBB including tight junction proteins (e.g. claudin-5, occludin), the glucose transporter Glut-1, and the efflux transporter p-gp/*MDR1* (termed hPSC-derived BMECs). Acquisition of these properties in the endothelium occurred in concert with translocation of β-catenin to the nucleus, suggesting an onset of Wnt-mediated signaling similar to *in vivo* studies [6,8]. Interestingly, glial fibrillary acidic protein^+^ (GFAP^+^) astrocytes and α-SMA^+^ pericytes/smooth muscle cells were detected at less than 1% of the total population and thus were not likely major contributors to the onset of BBB properties. Selective expansion in an endothelial cell growth medium based on formulations normally used for primary BMEC culture further enhanced the BBB phenotype in terms of Glut-1 expression level, while treatment with soluble inhibitors of Wnt signaling partially disrupted the acquisition of the BBB phenotype, indicating the potential contribution of neural cell-derived Wnts to this *in vitro* differentiation process. Interestingly, inhibition of Wnt signaling did not disrupt tight junction formation, which agrees with *in vivo* observations that endothelial-specific β-catenin knockout mice exhibit CNS hemorrhage but still possess BMECs expressing occludin and claudin-5 [6], and indicates that Wnt/β-catenin signaling is not the exclusive pathway regulating hPSC-derived BMEC formation [15-17]. Overall, these results demonstrate that endothelial cells having BBB properties can be obtained from primitive endothelium derived from hPSCs in a process that may mimic certain aspects of *in vivo* development.

These studies summarize the current use of stem cell sources for modeling BBB development. Stem cells offer many advantages over primary cells for studying development *in vitro*. For one, cellular yields are inconsequential when using stem cells due to the ability to scale undifferentiated cell populations, whereas primary embryonic sources of endothelial cells and particularly BMECs are nearly impossible to obtain in significant amounts. Another benefit is the ability to use human cells without needing access to scarce primary human tissue resources. In addition, while we and others have routinely used primary adult BMECs or cell lines to investigate the BBB induction process, this practice is largely flawed because in these cases one must combat an *in vitro* de-differentiation artifact, which does not necessarily correlate to induction and maintenance through a developmental pathway as one would expect with stem cell-based methods. This reasoning does not imply that all molecular and cellular studies using adult BMECs to model BBB induction are without merit; but instead, emphasizes that care must be exercised to interpret results obtained by the model in the appropriate context. Lastly, hPSC-derived BMECs could potentially be used to screen for developmental mechanisms and pathways relevant to BBB induction, as demonstrated by the observation that Wnt/β-catenin signaling affects acquisition of BBB properties. However, similar to the cautions described above for primary or cell line systems, care must be taken in the interpretation of such results and assumptions of *in vivo* relevance. For instance, *in vitro* differentiation may not fully recapitulate *in vivo* development if important molecular cues are absent or introduced at a time point where the hPSC-derived BMECs are not receptive to the cues. In our hPSC study, IMR90-4 and DF19-9-11T hiPSCs could be differentiated to pure populations of BMECs, but H9 hESCs generated a mixture of BMECs and non-BBB endothelium [96], presumably due to the reasons listed above. Similarly, other cues that are not typically present during *in vivo* BBB development could potentially induce BBB properties through a pathway distinct from that followed in normal development. Therefore, it would be advisable to use stem cell-derived BBB models as a complement, but not a replacement, for existing *in vivo* approaches such as transgenic animal models. Researchers are also becoming increasingly aware that heterogeneity in the brain is encoded during embryonic development [102-104] and the signals that govern this development may also contribute directly to patterns of brain vascularization and acquisition of BBB properties [105]. Therefore, NPCs isolated as bulk cortical populations and hPSCs differentiated to heterogeneous neural cells are unlikely to capture this diversity. Recent evidence has also suggested BBB heterogeneity in adult brain vessels at potentially single cell levels [106]. As such, future studies to determine the extent of hPSC-derived BMEC heterogeneity may also be an important consideration.

### Stem cell modeling of BBB maintenance and regulation

While modeling BBB development requires embryonic neural cells and immature BMECs, modeling adult BBB maintenance requires mature BMECs along with co-cultured cells of the adult neurovascular unit such as pericytes, astrocytes, and neurons (Figure 1). Unfortunately, adult BMECs and co-cultured cells are most often isolated from non-human sources, are generally acquired in low yield, are heterogeneous between isolations, and de-differentiate upon extended culture [107-109]. Stem cells could therefore also be an attractive alternative for adult BBB modeling.

To date, we have investigated using stem cells to replace primary neurons and astrocytes in *in vitro* co-culture models [66]. In this study, rat NPCs were differentiated under several different conditions to produce mixtures of neurons, astrocytes, oligodendrocytes, and proliferating neural progenitors (Figure 4b). The critical phenotype evaluated in this case was the capability of NPC-derived cell mixtures to induce TEER in cultured adult rat BMECs. By tuning differentiation time and medium composition, NPCs were differentiated to a mixture consisting predominantly of GFAP^+^/nestin^+^ astrocytes and nestin^+^/GFAP^-^/βIII tubulin^-^ progenitors that could effectively induce TEER compared to mixtures containing βIII tubulin^+^ neurons as the major population. Furthermore, NPCs differentiated for extended periods of time (12 days vs. 6 days) were more effective for TEER induction. With longer differentiation time, astrocytes acquired multiple extended processes indicative of physical maturation, which may contribute to their regulation of BBB phenotype. NPCs also exhibit a stable transcriptome after extended proliferation in the undifferentiated state [110], and accordingly, the ability of differentiated NPCs to upregulate TEER was unchanged between freshly isolated and extensively passaged NPCs, indicating the NPCs could be expanded to large yields without adverse effects on BBB induction. In addition to TEER, differentiated NPCs also regulated p-gp activity, tight junction fidelity in terms of continuous intercellular localization, and expression of various genes in a manner similar to primary astrocytes. Finally, these general strategies were adapted for *human* NPCs, and mixtures of astrocytes and neurons derived from human NPCs could similarly upregulate TEER in cultured rat BMECs, indicating NPCs could also be useful for human BBB modeling applications.

To further facilitate studies of human BBB maintenance and regulation, we developed a protocol for purifying the immature hPSC-derived BMECs described earlier, and used these cells to model the mature BBB (Figure 5) [96]. Facile purification of the hPSC-derived BMECs by passaging the mixed differentiated cultures, consisting of endothelial and neural cell types, onto collagen IV/fibronectin matrix yielded purified endothelial cell monolayers that when co-cultured with primary rat astrocytes possessed substantial barrier properties (maximum TEER achieved = 1450 Ωxcm^2^; average TEER over 30 independent differentiation and purification experiments = 860 ± 260 Ωxcm^2^), far exceeding reported values for primary cell and cell line-based human BBB models [41,48]. In addition, during the purification process the cells matured from a vascular perspective gaining VE-cadherin and vWF expression, and could uptake acetylated low-density lipoprotein and form vascular tubes upon VEGF stimulation. These hPSC-derived BMECs also expressed transcripts encoding a number of receptors and transporters found at the BBB *in vivo*, including nutrient receptors, amino acid and peptide transporters, and efflux transporters. Moreover, the efflux transporters were shown to possess functionally polarized activity similar to other primary models [96]. While the hPSC-derived model possesses favorable barrier characteristics compared to other human models, several pertinent questions need to be addressed to determine if hPSC-derived BMECs truly represents the “adult” BBB phenotype. For instance, despite elevated TEER (800–1000 Ωxcm^2^), the hPSC-derived BMECs still possess inferior barrier properties compared to the *in vivo* BBB (measured up to ~6000 Ωxcm^2^ in rats [21]). Along these lines, hPSC-derived BMECs do not encounter pericytes during the co-differentiation process [96], whereas pericytes contribute substantially to BBB development *in vivo*[7,111]. As such, optimization of hPSC-derived BMEC differentiation through discovery of other important BBB inductive factors and employment of additional co-culture schemes will likely be necessary to more fully reconstitute BBB properties. In addition, as has recently been performed for primary cultured BMECs [38,112] and the hCMEC/D3 line [113-115], transcriptome, proteome, and functionality tests will be required to determine how closely these cells resemble their *in vivo* counterparts and to determine which types of BBB studies are best supported by the hPSC-derived BBB model. To enhance BBB properties, components from each of the aforementioned stem cell modeling strategies could be combined to form a more accurate *in vitro* model. Human NPC-derived astrocytes and neurons (Figure 4b), for instance, could be utilized for co-culture with hPSC-derived BMECs. hPSCs have also been differentiated to astrocytes that exhibit some broad positional identity (e.g. dorsal vs. ventral and forebrain vs. hindbrain) [116,117], and these cells could be used to probe potential differences in region-specific BBB induction and maintenance. Along these lines, certain neurogenic regions of the adult brain may rely on interactions between the resident NPC population and brain vasculature to maintain NPC stemness and regulate the local barrier properties of the endothelium [118]. Thus, a combination of hPSC-derived BMECs and hPSC-derived NPCs [119] could potentially be used to model these complex interactions. In addition to brain cells, vascular cells with putative pericyte identity have also been differentiated from hPSCs [120,121]. Overall, hPSCs constitute a single cell source from which all components of an adult BBB model could in principle be obtained (Figure 5), pending advances in hPSC differentiation procedures to more appropriately capture the phenotype of each mature cell in the neurovascular unit. However, extensive characterization of each type of cell would be required to qualify these cell sources for BBB modeling.

One area where hPSCs have a clear advantage over primary cells and cell lines is in the modeling of diseases having a genetic component. Whereas primary diseased brain tissue is extremely heterogeneous and difficult to obtain from humans, hiPSC lines can be created directly from patients and then differentiated to the cell types of interest in high yield (Figure 5). Therefore, BBB models constructed from hiPSC-derived progeny may have future utility for understanding the genetic contributions of components of the neurovascular unit to complex CNS diseases. For instance, a recent study has identified the mechanism by which an isoform of apolipoprotein E (ApoE) contributes to neurodegeneration in Alzheimer’s disease and demonstrated that vascular defects precede the neurodegenerative disease phenotype [122]. Therefore, hiPSCs could be generated from Alzheimer’s patients carrying familial mutations that promote the disease phenotype [90], and these hiPSCs could be differentiated to both neurons and BMECs to study the effects of ApoE isoforms on disease progression within the neurovascular unit *in vitro* using human cells. In general, as genetic, epigenetic, and environmental causes of other neurological diseases become better understood, hiPSCs could be used to capture the dynamics of disease progression and cell-cell interactions *in vitro*.

### Stem cell models for drug screening applications

As previously discussed, a major motivation for designing an *in vitro* BBB model is the capability to assess drug delivery potential of candidate therapeutics. *In vitro* models using BMECs of non-human origin are most widely used for drug screening [123-125]. Moreover, the hCMEC/D3 line constitutes the only human brain endothelial cell line widely available for larger scale screening studies. Although these and other immortalized human cell lines may have some potential for assessing drug substrate potential for the various efflux transporters, their usage for drug screening applications remains suboptimal due to low TEER values and relatively high basal permeability [41].

The use of purified hPSC-derived human BMECs may represent an alternative cell source for human BBB drug screening [96]. As mentioned previously, while hPSC-derived BMEC monocultures have reasonable baseline TEER values (~250 Ωxcm^2^), they can achieve up to 1450 Ωxcm^2^ after medium and astrocyte co-culture optimization. This model demonstrated lower permeability to sucrose (P_*e*_ = 3.4 × 10^-5^ cm/min) than those values published on hCMEC/D3 monolayers (1.65 × 10^-3^ cm/min) [41] or bovine BMEC/astrocyte co-cultures (0.75 × 10^-3^ cm/min) [123]. In addition to low sucrose permeability, hPSC-derived BMECs co-cultures exhibited a 40-fold range in permeability between diazepam (BBB permeable) and sucrose (BBB impermeable) compared with the 10-fold and 20-fold ranges reported for hCMEC/D3 and bovine BMECs, respectively [41,123]. In addition, a small cohort of molecules, including substrates of influx and efflux transport, was analyzed for permeability across the hPSC-derived *in vitro* BBB model. The resultant permeability values correlated well with *in vivo* uptake measured by *in situ* perfusion in rodents. Another important standard for an *in vitro* BBB model suitable for drug screening is the expression and polarized activity of efflux transporters. Efflux transporters constitute a major challenge for drugs that may present a low permeability despite having the desirable size and lipophilic properties. Three members of the ABC transporters that mediate much of the efflux activity at the BBB are p-gp (*MDR1*/*ABCB1*), MRPs (*ABCC*s) and BCRP (*ABCG2*). hPSC-derived BMECs were found to express p-gp, MRP-1, MRP-2, MRP-4, and BCRP transcripts, and p-gp protein expression was validated using immunocytochemistry [96]. Functional activity of these transporters was confirmed using Rhodamine 123 and doxorubicin as substrates in both accumulation and permeability assays. We noted a 2.3-fold increase in trans-BBB transport for the p-gp substrate, Rhodamine 123, following p-gp inhibition by cyclosporin A (CsA). Similar efflux inhibition results were noted with the pan-substrate doxorubicin following inhibition with CsA, Ko143 (BCRP inhibitor), or MK571 (pan-MRP inhibitor). The hCMEC/D3 cell line yields comparable efflux inhibition values [126], but a larger, 3-fold change in brain uptake of Rhodamine 123 is observed in rodents upon p-gp inhibition [127]. Activity of these transporters was also implicit by relative permeability measurements, where colchicine, vincristine, and prazosin (substrates recognized by various ABC transporters) exhibited lower apical-to-basolateral permeability than their relative lipophilicity would suggest.

In addition to drug permeability screening and efflux transporter assessment, hPSC-derived BMECs could serve as a useful tool for evaluation of solute carriers, receptors involved in receptor-mediated endocytosis and transcytosis processes, or screening for BBB targeting reagents. For example, the hPSC-derived BMECs express transcripts encoding several solute carriers recognized as enriched at the BBB such as Glut-1 (*SLC2A1*), large neutral amino acid transporter-1 (*SLC7A5*), monocarboxylate transporter-1 (*SLC16A1*) and system N amino acid transporter-5 (*SLC38A5*) [96]. Furthermore, the hPSC-derived model appeared devoid of Oatp14 (*SLCO1C1*) transcript, an organic anion transporter that is highly expressed in rodents, but not humans [128,129], suggesting at least a limited level of species restricted expression. We also reported transcript expression for several receptors involved in receptor-mediated transport such as insulin receptor, leptin receptor, and transferrin receptor.

Ultimately, more extensive work will be necessary to determine the full utility of hPSC-derived BMECs for drug screens. For example, seven compounds were tested in the original hPSC-derived BMEC model as a proof of concept study, but this amount is by no means exhaustive enough to determine its true predictive power. Therefore, it would be advisable to test a larger compound library. In addition, various transporters were assayed at the transcript level and some at the protein and functional levels. However, similar to other *in vitro* models built on primary or cell line-based BMECs, it is unlikely that hPSC-derived BMECs will ever fully mimic the transcriptome and proteome of the *in vivo* BBB. Thus, comparative analyses using techniques such as quantitative mass spectrometry and microarray or RNAseq would be useful to determine both advantages and shortcomings of these cells. Such data would also likely yield molecular targets and pathways that need to be modulated to achieve a screening platform more representative of the *in vivo* BBB.

Finally, the choice of hPSC line may affect the predictive nature of the resultant BMEC population. Line-to-line variability in differentiation efficiency is not uncommon when using hESCs or hiPSCs [130,131], and in our experience, while each of the lines produced cells that expressed BMEC markers in the mixed differentiating cultures, the functional properties of the purified BMEC population varied. It is interesting to note that different hiPSC reprogramming methods and donor fibroblast sources yielded purified BMECs having barrier phenotypes. For example, IMR90-4-derived hiPSCs were reprogrammed from fetal lung fibroblasts using retroviral transduction and DF19-9-11T hiPSCs were reprogrammed from foreskin fibroblasts by non-integrating episomal vectors. In contrast, the DF6-9-9T line, which was derived in the same study as the DF19-9-11T line, did not produce cells that generated a significant barrier phenotype following the identical differentiation protocol. Furthermore, the H9 hESC line generated a mixture of BMECs and non-BBB endothelium with this protocol. While we have not yet explored the possibility, it may also be possible that BMEC properties could be affected by the type of reprogrammed somatic cell (i.e. reprogrammed fibroblasts vs. neurons vs. endothelial cells, etc.) or the individual donor as some studies have shown that hiPSCs or cells differentiated from hiPSCs retain an epigenetic memory of their cell type of origin [132-134] or donor [135] following reprogramming. Overall, the results from the initial hPSC study indicate the BMEC differentiation protocol may have to be optimized and validated for individual lines. Although methodological enhancements are sure to improve the line-to-line consistency in BMEC production, we would currently recommend using the IMR90-4 hiPSC line as this line has been the most extensively validated in our hands. Importantly, once a line is validated, it is a highly scalable source of BMECs: by simply expanding cells in the undifferentiated hPSC stage, one can generate enough hPSC-derived BMECs for tens of thousands of Transwell filters from a single vial of stem cells. Overall, while we are highly encouraged by the properties of this first generation hPSC-derived BBB model, including its phenotype, yield, and scalability, more extensive characterization is warranted to test its utility for predictive drug screening applications.

## Conclusions

Stem cells have proven useful over the last decade for modeling various developmental and disease processes in humans. They have also provided access to unlimited quantities of differentiated human cells that are otherwise difficult or impossible to acquire. Based on the properties of hPSC-derived BMECs, and the lack of existing human BMEC sources, a stem cell model of the BBB could have significant impact on studies of BBB development and maintenance as well as for drug screening applications. The hPSC-derived BMECs could also be employed in BBB model formats that better mimic the physiological microenvironment, such as in matrices that enable the assembly of three-dimensional vascular structures [62] or systems that incorporate fluid flow [136]. Such improvements may further increase the relevance of mechanistic studies of the neurovascular unit or improve the predictive power of drug screens.

Looking beyond the traditional uses for BBB models, the capability to generate hiPSCs from patient-derived materials offers an unexplored niche for stem-cell derived BBB modeling. For instance, skin cells could be biopsied from patients and control groups, reprogrammed to pluripotent stem cells using any number of hiPSC derivation techniques, and differentiated to provide an isogenic supply of BMECs and neural cells to conduct CNS disease studies *in vitro*. Furthermore, advances in the genetic manipulation of hPSCs using tools such as bacterial artificial chromosomes [137], zinc finger nucleases [138], and TAL effector nucleases [139] could allow genetic manipulation akin to transgenic animal models to explore open-ended hypotheses regarding cell-specific and genetic contributions to disease states. While these strategies will likely always require an *in vivo* complement to verify experimental outcomes, they could substantially shorten exploratory endeavors and translate outcomes observed in animal studies to human cells. Given that hPSC culture techniques are becoming increasingly simplified with defined medium and matrix components that do not require feeder cells [140] and that the availability of hPSC lines is rapidly expanding via nonprofit centers such as the American Type Culture Collection (ATCC), the WISC Bank at the WiCell Research Institute, and the Harvard Stem Cell Institute, it should be possible for researchers to readily apply these techniques in future BBB studies.

### Statement of institutional approval

All studies described in this review were conducted according to policies set forth by the University of Wisconsin-Madison.

## Abbreviations

BBB: Blood–brain barrier; NPC: Neural progenitor cell; hPSC: Human pluripotent stem cell; hESC: Human embryonic stem cell; hiPSC: Human induced pluripotent stem cell; CNS: Central nervous system; ECM: Extracellular matrix; BMEC: Brain microvascular endothelial cell; EC: Endothelial cell; PNVP: Perineural vascular plexus; VEGF: Vascular endothelial derived growth factor; Shh: Sonic hedgehog; TJ: Tight junction; TEER: Transendothelial electrical resistance; p-gp: p-glycoprotein; MRP: Multi-drug resistance-associated protein; BCRP: Breast cancer resistance protein; vWF: Von Willebrand Factor; GFAP: Glial fibrillary acidic protein; ApoE: Apolipoprotein E; CsA: Cyclosporin A.

## Competing interests

The authors have filed several patent applications dealing with technology reviewed in this manuscript.

## Authors’ contributions

ESL, A A-A, SPP, and EVS wrote the manuscript. All authors have read and approved the final version of the manuscript.
